# CD4^+^ Natural Regulatory T Cells Prevent Experimental Cerebral Malaria via CTLA-4 When Expanded In Vivo

**DOI:** 10.1371/journal.ppat.1001221

**Published:** 2010-12-09

**Authors:** Ashraful Haque, Shannon E. Best, Fiona H. Amante, Seri Mustafah, Laure Desbarrieres, Fabian de Labastida, Tim Sparwasser, Geoffrey R. Hill, Christian R. Engwerda

**Affiliations:** 1 Immunology and Infection Laboratory, Queensland Institute of Medical Research and The Australian Centre for Vaccine Development, Herston, Brisbane, Queensland, Australia; 2 Institute of Infection Immunology, TWINCORE, Centre for Experimental and Clinical Infection Research (a joint venture between the Medical School Hannover (MHH) and the Helmholtz Centre for Infection Research (HZI)), Hannover, Germany; 3 Bone Marrow Transplantation Laboratory, Queensland Institute of Medical Research and The Australian Centre for Vaccine Development, Herston, Brisbane, Queensland, Australia; London School of Hygiene and Tropical Medicine, United Kingdom

## Abstract

Studies in malaria patients indicate that higher frequencies of peripheral blood CD4^+^ Foxp3^+^ CD25^+^ regulatory T (Treg) cells correlate with increased blood parasitemia. This observation implies that Treg cells impair pathogen clearance and thus may be detrimental to the host during infection. In C57BL/6 mice infected with *Plasmodium berghei* ANKA, depletion of Foxp3^+^ cells did not improve parasite control or disease outcome. In contrast, elevating frequencies of natural Treg cells in vivo using IL-2/anti-IL-2 complexes resulted in complete protection against severe disease. This protection was entirely dependent upon Foxp3^+^ cells and resulted in lower parasite biomass, impaired antigen-specific CD4^+^ T and CD8^+^ T cell responses that would normally promote parasite tissue sequestration in this model, and reduced recruitment of conventional T cells to the brain. Furthermore, Foxp3^+^ cell-mediated protection was dependent upon CTLA-4 but not IL-10. These data show that T cell-mediated parasite tissue sequestration can be reduced by regulatory T cells in a mouse model of malaria, thereby limiting malaria-induced immune pathology.

## Author Summary

Severe malaria can kill people via complications such as cerebral malaria. The number of malaria parasites in the body is a major determinant of whether a patient will develop severe disease. T cells are thought to help control parasite numbers, but regulatory T cells, which are known to dampen immune responses, are present at a greater frequency in the blood of malaria patients with the highest parasitemia, suggesting that these cells might impair parasite control. Our experiments in a mouse model of cerebral malaria show for the first time that regulatory T cells can contribute to protection against disease. Specifically, our data shows that accumulation of parasites in host tissues can be promoted by anti-parasitic T cell responses, and that regulatory T cells can reduce this parasite tissue sequestration and protect against experimental cerebral malaria if their numbers are sufficiently elevated. These results suggest that regulatory T cells can help reduce pathogenic T cell responses during experimental infection and protect against malaria induced immune pathology.

## Introduction

Severe malaria syndromes, including cerebral malaria (CM), claim the lives of approximately 900,000 people annually, mostly children under the age of 5 living in sub-Saharan Africa [1]. The mechanisms of CM pathogenesis remain poorly understood, since studies in humans are often restricted to post-mortem examinations. In particular, the roles played by the host immune response in either driving or preventing CM are unclear. It is possible that the immune response could be over-exuberant in some CM patients or lethargic in others, the balance of which may depend on the patient's and the parasite's genetic background. Several studies in malaria patients have reported associations between higher frequencies of peripheral blood regulatory T (Treg) cells and increased parasitemia [2], [3], [4]. However, these studies provided limited mechanistic insight into the role of Treg cells in severe malarial disease.

Under homeostatic conditions, Treg cells limit potentially aberrant T cell responses, thus preventing autoimmunity [5]. However, they can also impair effective pathogen clearance [6], [7], [8], while potentially playing a beneficial role in preventing immune-pathology during infection. The molecular mechanisms by which Treg cells perform these functions are incompletely understood, but have been reported to involve production of cytokines such as TGFβ and IL-10, and increased expression of the negative regulatory molecule CTLA-4 [9], [10], [11]. Furthermore, it is not known whether Treg cells act directly upon conventional T cells or on accessory cells such as antigen-presenting cells. Nevertheless, Treg cells are often viewed as detrimental during infection, since they may impede the generation of effective pathogen-specific T cell responses. Thus, an emerging paradigm is that Treg cells block T cell-mediated clearance of malaria parasites in humans, facilitating an increase in parasitemia.

The direct study of immune mechanisms in malaria patients is problematic for obvious practical and ethical reasons. Therefore, mouse models of severe and non-severe malaria have been employed to study the immune response to infection. Studies in an experimental model of cerebral malaria (ECM) caused by infection of C57BL/6 mice with *P. berghei* ANKA (*PbA*) have highlighted the important role played by various immune cells in disease pathogenesis, including CD4^+^ T cells, CD8^+^ T cells, conventional dendritic cells and Natural Killer (NK) cells [12], [13], [14], [15], [16], [17], [18]. In mice that succumb to ECM, parasite biomass is poorly controlled and there is clear evidence of immune-mediated parasite tissue sequestration [19]. Until recently, the deleterious role proposed for Treg cells in studies of human malaria has been difficult to test in mice, due to the lack of appropriate reagents [20], [21]. Our initial studies indicated a detrimental role for Treg cells because depletion of CD25^hi^ cells prior to infection, the majority of which were Treg cells, protected mice from ECM and was associated with increased antigen-specific CD4^+^ T cell responses [20]. Recently however, specific depletion of FoxP3^+^ Treg cells did not protect against ECM, bringing into question the role for these cells in mediating disease [22]. Although the effect of Treg cell depletion on T cell responses and pathogen burden was not studied [22], given that ECM is mediated by pathogenic T cells that promote parasite tissue sequestration [19], we hypothesized that under certain conditions, Treg cells can suppress deleterious T cell responses and protect against ECM.

One approach to manipulate Treg cell numbers in vivo has been to use IL-2/anti-IL-2 antibody complexes to potentiate IL-2 signalling and drive expansion of FoxP3^+^ Treg cells [23]. Certain monoclonal antibodies (mAbs) bind to IL-2 in such a way that its signalling capacity is preserved, while its in vivo half-life is dramatically extended [24]. Moreover, different mAbs against IL-2 bind to different regions of the molecule, thus skewing its signalling capacity [25]. For example in mice, IL-2 bound to S4B6 mAb is not capable of interacting with the high affinity, heterotrimeric IL-2 receptor, but does interact with the lower affinity heterodimeric receptor. In contrast, IL-2 bound to JES6-1A12 mAb retains the ability to interact with the higher affinity receptor. IL-2/anti-IL-2 complexes profoundly alter lymphocyte dynamics during homeostasis, autoimmunity and vaccination [23], [25], [26], [27], [28]. Recently, IL-2/JES6-1A12 was shown to expand Treg populations, prevent auto-immunity and induce long term graft tolerance [23]. Here, we show for the first time that while removal of naturally-occurring Treg cells minimally affects the course of disease, increasing their numbers in vivo throughout the course of infection via IL-2/anti-IL-2 antibody complexes allows these cells to protect against ECM.

## Results

### Total Foxp3^+^ cell depletion has minimal effects on pathogen burden, T cell responses or clinical outcome in ECM

**Figure 1:**
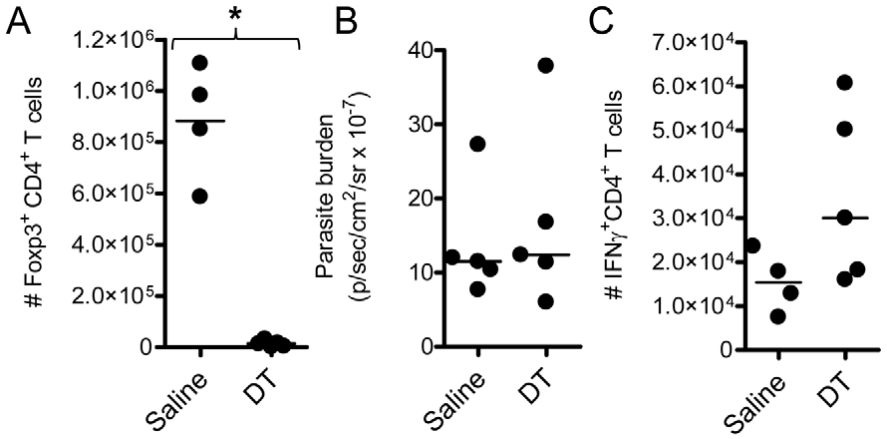
Total Foxp3^+^ cell depletion does not affect parasite burden or disease outcome in ECM. DEREG mice (n = 5) were treated with DT (1µg i.p.) or saline on the day prior to infection, and on days 2 and 4 after infection with *Pb*A. On day 4 p.i., A) Foxp3^+^ cell depletion and C) the magnitude of the IFNγ CD4^+^ T cell response was determined in the spleen by flow cytometry. B) Whole body parasite burdens were assessed on day 6 p.i.. These data are representative of 2 independent experiments. Mann-Whitney: *p<0.05.

The *foxp3*-DTR transgenic (DEREG) mouse was recently used to deplete Foxp3^+^ cells prior to and over the course of *Pb*A infection, with no impact on susceptibility to ECM [22]. We employed the same system here to study the effect of Foxp3^+^ cell depletion on T cell responses and pathogen burden during ECM. Consistent with the published data, in our hands DEREG mice depleted of Foxp3^+^ cells the day prior to, and over the course of infection (Figure 1A), remained as susceptible to ECM as Foxp3^+^ cell replete DEREG mice (data not shown). Furthermore, we observed no change in whole body parasite burden (Figure 1B); with a trend towards an increase in the splenic IFNγ^+^ CD4^+^ T cell response (Figure 1C). These data show that the depletion of Foxp3^+^ cells had little effect on pathogen burden or disease outcome during ECM.

### IL-2/anti-IL-2 complexes protect against ECM

**Figure 2:**
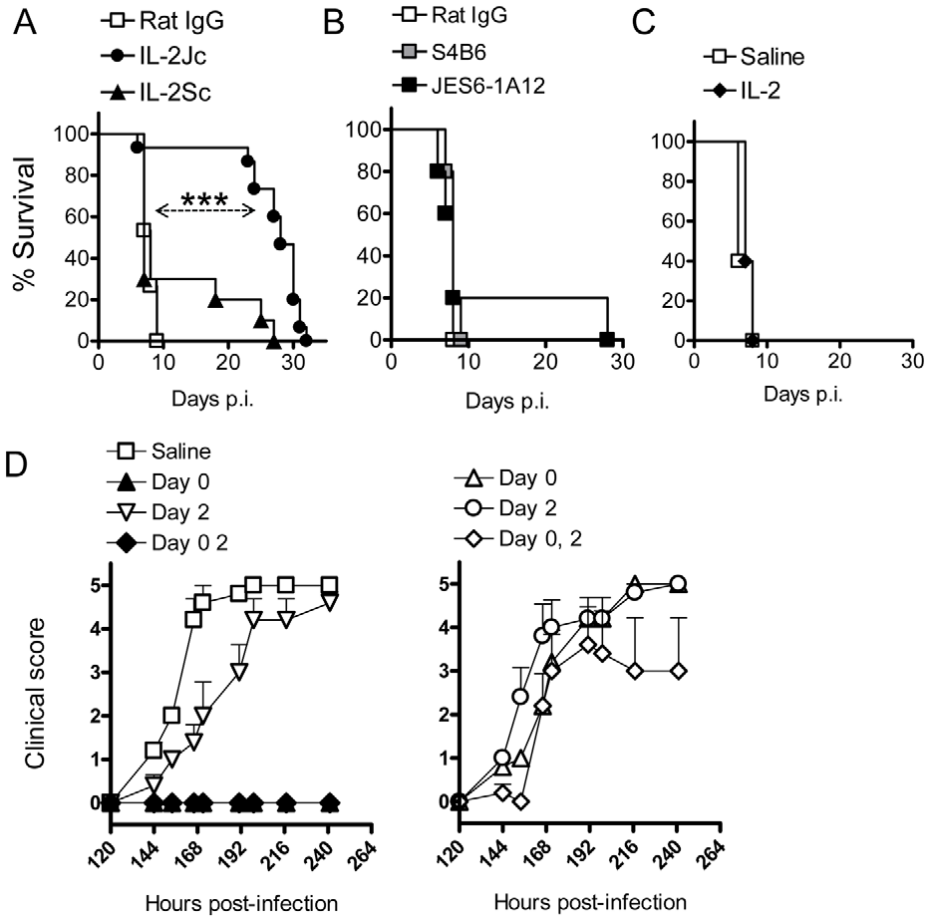
IL-2/anti-IL-2 complex protects against ECM. A) C57BL/6 mice (n = 10–15) were infected with *Pb*A, and treated on day 0 p.i. with complexes IL-2Jc, IL-2Sc, or with control rat IgG. Mice were monitored for survival. Log Rank: ***p<0.001. This experiment is representative of >5 independent experiments. B) C57BL/6 mice (n = 5), infected as above, were treated i.p. with 0.5mg of S4B6, JES6-1A12 or control mAb on days 0 & 3 p.i., and monitored for survival. These data are representative of 2 independent experiments. C) C57BL/6 mice (n = 5), infected as above, were treated i.p. with 1.5µg of recombinant mouse IL-2 on the day of infection and monitored for survival. These data are representative of 2 independent experiments. D) Graphs illustrate data from a single representative experiment of 2 in which infected mice were treated i.p. on days indicated with either a standard dose of IL-2Jc (left graph) or a 10-fold lower dose (right graph). Mice were scored for clinical symptoms from day 5 (120 hours p.i.).

Since removal of Foxp3^+^ cells had no effect on disease progression, we next examined whether increasing numbers of Foxp3^+^ cells would impact upon ECM development. Therefore C57BL/6 mice were infected with *Pb*A and immediately treated with a single dose of IL-2/JES6-1A12 (hereafter referred to as IL-2Jc) or IL-2/S4B6 (IL-2Sc) complexes. The IL-2Jc complex binds the high affinity heterotrimeric IL-2 receptor to drive Treg cell expansion, while the IL-2Sc complex binds the lower affinity heterodimeric IL-2 receptor resulting in the expansion of activated CD8^+^ T cells and NK cells [25]. Control mice that received rat IgG displayed clinical signs of illness from day 6 post-infection (p.i), and succumbed to infection with neurological symptoms typical of ECM with a Median Survival Time (MST) of 8 days (Figure 2A). Mice treated with IL-2Sc were also susceptible to ECM (MST: 7 days), demonstrating that IL-2Sc afforded no protection against infection (Figure 2A). In stark contrast, IL-2Jc treated, infected mice, rarely displayed ECM symptoms and were protected from ECM-related morbidity, dying instead from hyperparasitemia with an MST of 28 days (Figure 2A). Mice treated with S4B6 alone, JES6-1A12 alone or recombinant IL-2 alone, were as susceptible to ECM as control mice (Figures 2B & 2C). Together, these data demonstrate a specific capacity for IL-2Jc, but not its component parts in isolation or an alternative IL-2 antibody complex (IL-2Sc), to protect against ECM.

To investigate the timing and dosing requirements for IL-2Jc-mediated protection, *Pb*A-infected mice were treated on days 0 or 2 p.i., or on both days with either a standard IL-2Jc dose (1.5ug cytokine: 50ug antibody) (Figure 2D left) or a ten-fold lower dose (Figure 2D right). Control mice displayed clinical signs of disease from day 6 p.i., with 100% of mice succumbing to ECM by day 8 p.i. Only mice that had received a standard IL-2Jc dose on day 0 were protected from ECM. Importantly, mice receiving a delayed IL-2Jc dose on day 2 p.i. were completely susceptible to ECM. Thus, IL-2Jc protects against ECM only when administered at the time of infection.

### IL-2Jc prevents the rapid increase in parasite burden associated with ECM

**Figure 3:**
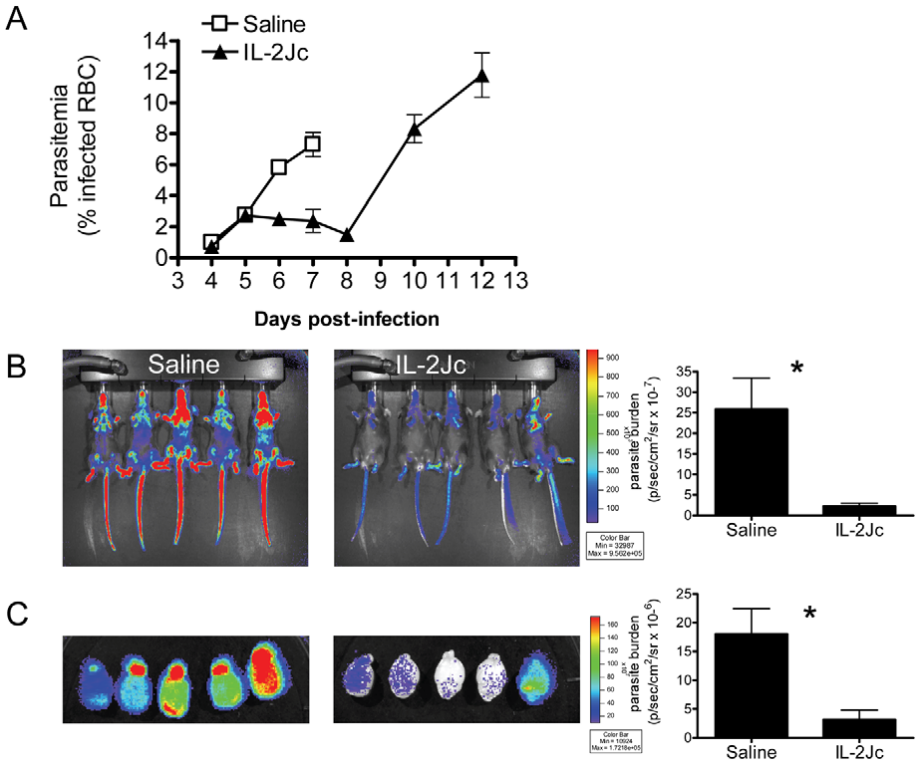
IL-2Jc treatment reduces parasite biomass during ECM. A) Peripheral blood parasitemia was determined in IL-2Jc-treated and control treated mice throughout *Pb*A infection. Control mice assessment was stopped when mice began to succumb to infection. B) C57BL/6 mice were infected with transgenic *Pb*A-luc, expressing luciferase, and i.p. treated with IL-2Jc or control saline on the same day. On day 6 p.i., when control mice were exhibiting symptoms of ECM, mice were imaged for parasite-derived bioluminescence. Rainbow coloured images depict increasing intensities of light, from purple to red. Data is summarised on the accompanying graph. C) Perfused brains from mice in B) were also assessed for parasite biomass; Mann-Whitney: *p<0.05. These data are representative of 3 independent experiments.

C57BL/6 mice typically display ECM symptoms when blood parasitemia reaches ∼7–10% parasitized red blood cells (pRBCs) (Figure 3A). Blood parasitemia in IL-2Jc-treated mice was similar to control, infected mice on days 4 & 5 p.i. (Figure 3A). However, from day 6 p.i. onwards, when clinical symptoms appeared in control mice, IL-2Jc treated mice displayed significantly lower blood parasitemia for the following 3 days, only rising again from day 10 p.i. onwards (Figure 3A). While blood parasitemia has been routinely used to monitor disease progression, it is now recognised that measurements of total parasite biomass in the whole body offer a better correlate of the disease status of malaria patients [29]. To assess parasite biomass in infected mice, we used a transgenic *Pb*A strain engineered to constitutively express firefly luciferase (*Pb*A-luc) [20]. The bioluminescence generated by *Pb*A-luc parasites at any given time is directly proportional to the sum of parasites in the tissues and circulating blood of the infected animal [19], [20], [30] (Figure 3B). IL-2Jc-treated, *Pb*A-luc-infected mice harboured significantly lower parasite biomass compared to control mice on day 6 p.i., when control animals displayed severe ECM symptoms. Moreover, following whole body perfusion to remove circulating RBCs, brains from IL-2Jc-treated mice also exhibited significantly lower parasite sequestration than brains from control animals (p<0.05) (Figure 3C). These data demonstrate that IL-2Jc-mediated protection against ECM was associated with lower parasite biomass and reduced pRBC brain sequestration.

### Effect of IL-2Jc on recruitment of leukocytes to the brain

**Figure 4:**
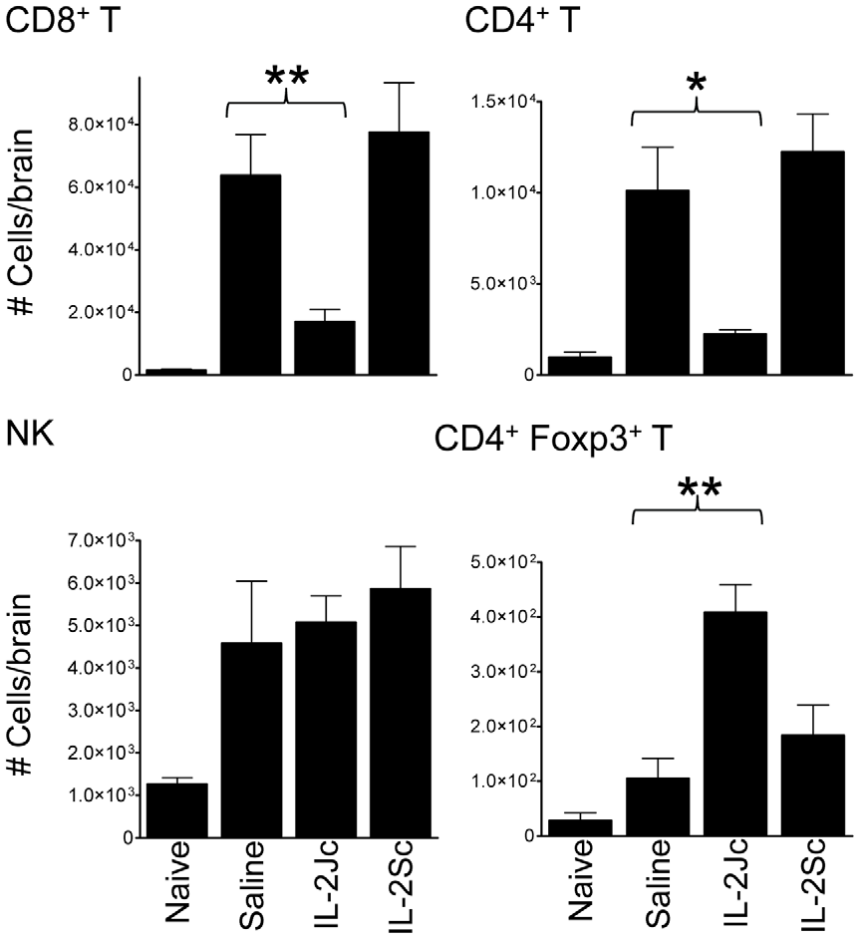
IL-2Jc blocks conventional T cell recruitment to the brain during ECM. Brain sequestered leukocytes from naïve, *Pb*A-infected & saline treated, and *Pb*A-infected, IL-2Jc or IL-2Sc-treated C57BL/6 mice (n = 5) were isolated on day 6 p.i., when control, infected mice were displaying ECM symptoms, and phenotyped by flow cytometry. Mann-Whitney: *p<0.05; **p<0.01. These data are representative of 3 independent experiments.

ECM is associated with the recruitment of CXCR3^+^ leukocytes to the brain [13], [31], [32], [33], [34], including T cells responsible for disease pathology [16], [18]. On day 6 p.i., the recruitment of CD8^+^ and CD4^+^ T cells, but not NK cells, to the brain was significantly reduced by IL-2Jc treatment, compared with mice receiving either IL-2Sc or control treatment (Figure 4). Furthermore, Treg cell numbers were significantly higher in IL-2Jc-treated mice compared to all other groups studied (Figure 4). These data indicated that IL-2Jc-mediated protection was associated both with a specific blockade of conventional T cell recruitment to the brain, and also an increase in the number of Treg cells in this tissue site.

### IL-2Jc–mediated protection is associated with suppression of effector CD8^+^ and CD4^+^ T cells, and expansion of Treg cells

**Figure 5:**
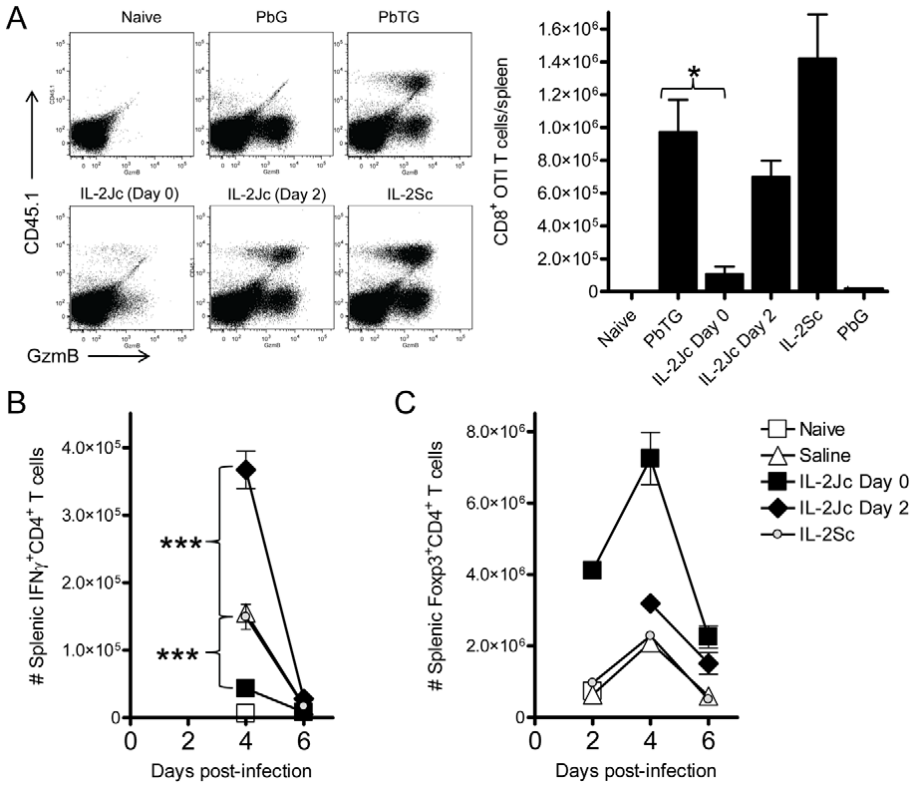
IL-2Jc-mediated protection is associated with impaired expansion of antigen-specific CD8^+^ T cells, reduced effector CD4^+^ T cell responses, and Treg cell expansion. A) C57BL/6 mice were adoptively transferred with 10,000 CD8^+^ CD45.1^+^ OTI T cells, and infected with SIINFEKL-expressing transgenic *Pb*TG. Control mice were infected with non-SIINFEKL-expressing *Pb*G parasites. *Pb*TG-infected mice were i.p. treated with IL-2Jc (day 0 or day 2), IL-2Sc (day 0), or control saline. On day 6 p.i., when control mice were displaying ECM symptoms, splenic CD8^+^ T cells (gated in FACS plots) were assessed for the presence of CD45.1^+^ SIINFEKL-specific T cells, and expression of the activation marker, GzmB. The graph indicates the number of CD45.1^+^ CD8^+^ SIINFEKL-specific T cells per spleen. Mann-Whitney * p<0.05. These data are representative of 2 independent experiments. B&C) C57BL/6 mice (n = 5) were infected with *PbA*, and i.p. treated with IL-2Jc (day 0 or day 2 p.i), IL-2Sc (day 0) or control saline. On days indicated, splenocytes were assessed directly ex vivo for the number of B) IFNγ-producing effector CD4^+^ T cells and C) CD4^+^ Foxp3^+^ Treg cells. Mann-Whitney: ***p<0.001. These data are representative of 2 independent experiments.

CD8^+^ T cells play a key role in ECM pathology [16], [18]. A previous study using a transgenic *Pb*A strain expressing model T cell epitopes showed that antigen-specific CD8^+^ T cells are primed in the spleen [35]. We employed this experimental system to assess the fate of antigen-specific CD8^+^ T cells in IL-2Jc-treated mice during ECM. OVA-specific, congenic (CD45.1) CD8^+^ T (OTI) cells were transferred into mice prior to infection with OVA-transgenic *Pb*A (*Pb*TG) or a non-OVA-expressing control parasite (*Pb*G). On day 6 p.i., splenic OTI cell numbers and activation status, via Granzyme B (GzmB) expression, were assessed (Figure 5A). OTI cells were not detected in the spleens of naïve mice or mice infected with *Pb*G (Figure 5A). The expression of GzmB was detected in ∼30% of endogenous (CD45.1 negative) CD8^+^ T cells in *Pb*G-infected mice, indicating dramatic activation of CD8^+^ T cells at the onset of ECM. In control treated mice infected with *Pb*TG, OTI cells were readily detected, indicating antigen-specific activation and proliferation of these cells had occurred. Moreover, nearly all of these cells expressed GzmB, at a level similar to activated endogenous CD8^+^ T cells. IL-2Jc treatment at the time of infection dramatically impaired, though did not abrogate, the OTI CD8^+^ T cell response (Figure 5A). This effect was not apparent in mice treated on day 2 p.i. with IL-2Jc. Furthermore, mice treated with IL-2Sc displayed a trend towards an enhanced OTI T cell response compared to control mice, which is consistent with reports of the stimulatory effect of IL-2Sc on CD8^+^ T cells [25], [26], [27], [28], [36]. Together these data demonstrate that IL-2Jc, when administered on the day of infection, potently inhibits pathogenic, antigen-specific CD8^+^ T cell responses during ECM.

To be sure that IL-2Jc did not stimulate NK cells or NKT cells, we examined their expression of the activation markers CD69, GzmB and IFNγ, 24 hours after infection and treatment with IL-2Jc. As expected, no further activation of NK or NKT cells in IL-2Jc-treated mice was detected relative to control-treated, infected mice; while in contrast, IL-2Sc-treatment clearly stimulated both NK and NKT cells (Figure S1A). Furthermore, neither depletion of NK cells with anti-NK1.1 antibody, nor the absence of invariant chain NKT cells in B6.Jα18^−/−^ mice, impeded either CD4^+^ Foxp3^+^ T cell expansion (Figure S1B) or control of parasite burden (Figure S1C) by IL-2Jc. Together these data indicate that IL-2Jc does not protect against ECM via activation of NK cells or NKT cells.

*Pb*A infection induces a potent pro-inflammatory cytokine response in C57BL/6 mice that is strongly associated with ECM pathogenesis. IFNγ is absolutely critical for disease onset [37], [38], [39], possibly by promoting *PbA* tissue sequestration [19]. We found that IL-2Jc administered on the day of infection resulted in lower serum IFNγ levels by day 4 p.i., whereas neither IL-2Sc nor delayed IL-2Jc treatment had any significant effect (Figure S2). Examination of the antigen-specific splenic CD4^+^ T cell response indicated an impaired ex vivo proliferative and IFNγ recall response from IL-2Jc treated mice (Figure S3), suggesting that in vivo CD4^+^ T cell responses were impaired by IL-2Jc treatment. Therefore, we enumerated splenic IFNγ-producing CD4^+^ T cells and Treg cells over the course of *Pb*A-infection in mice treated with IL-2Jc or IL-2Sc. IFNγ-producing CD4^+^ T cells were detectable from day 4 p.i. onwards in infected, but not naïve mice (Figure 5B). Control saline-treated and IL-2Sc-treated infected mice had very similar numbers of IFNγ^+^ CD4^+^ T cells on day 4 p.i.. In contrast, IL-2Jc treatment suppressed the number of IFNγ^+^ CD4^+^ T cells (p<0.001). Interestingly, the number of IFNγ^+^ CD4^+^ T cells was greatly enhanced if IL-2Jc was administered on day 2 p.i. (p<0.001)(Figure 5B), possibly indicating increased expression of high affinity IL-2 receptor on these cells by day 2 p.i.. Treg cells expanded in control, infected mice and numbers peaked on day 4 p.i., before declining by day 6 p.i. (Figure 5C), consistent with previous reports [20], [21], [40]. Infected mice treated with IL-2Sc exhibited almost identical Treg cell expansion kinetics to that of control treated mice (Figure 5C), consistent with the notion that IL-2Sc has little impact on Treg cell numbers [25]. Strikingly, IL-2Jc treatment at the time of *Pb*A infection resulted in a dramatic expansion in Treg cell numbers with a >6-fold increase by day 2 p.i., peaking at day 4 p.i. (∼3.5-fold greater numbers than in control treated mice), before retracting somewhat by day 6 p.i., although numbers still remained ∼4-fold greater than in IL-2Sc and control treated groups. In contrast, delaying IL-2Jc treatment until day 2 p.i., resulted in very little enhanced Treg cell expansion. Thus, the protection afforded by IL-2Jc treatment at the time of *Pb*A infection was associated with a dramatic and sustained elevation of Treg cell numbers over the course of infection. Taken together, these data show that IL-2Jc-mediated protection against ECM was associated with an expansion of CD4^+^ Treg cells and an accompanying impairment of the conventional CD8^+^ and CD4^+^ T cell responses.

### IL-2Jc treatment during ECM causes natural Treg cells to proliferate and express higher levels of Foxp3, IL-10 and CTLA-4

**Figure 6:**
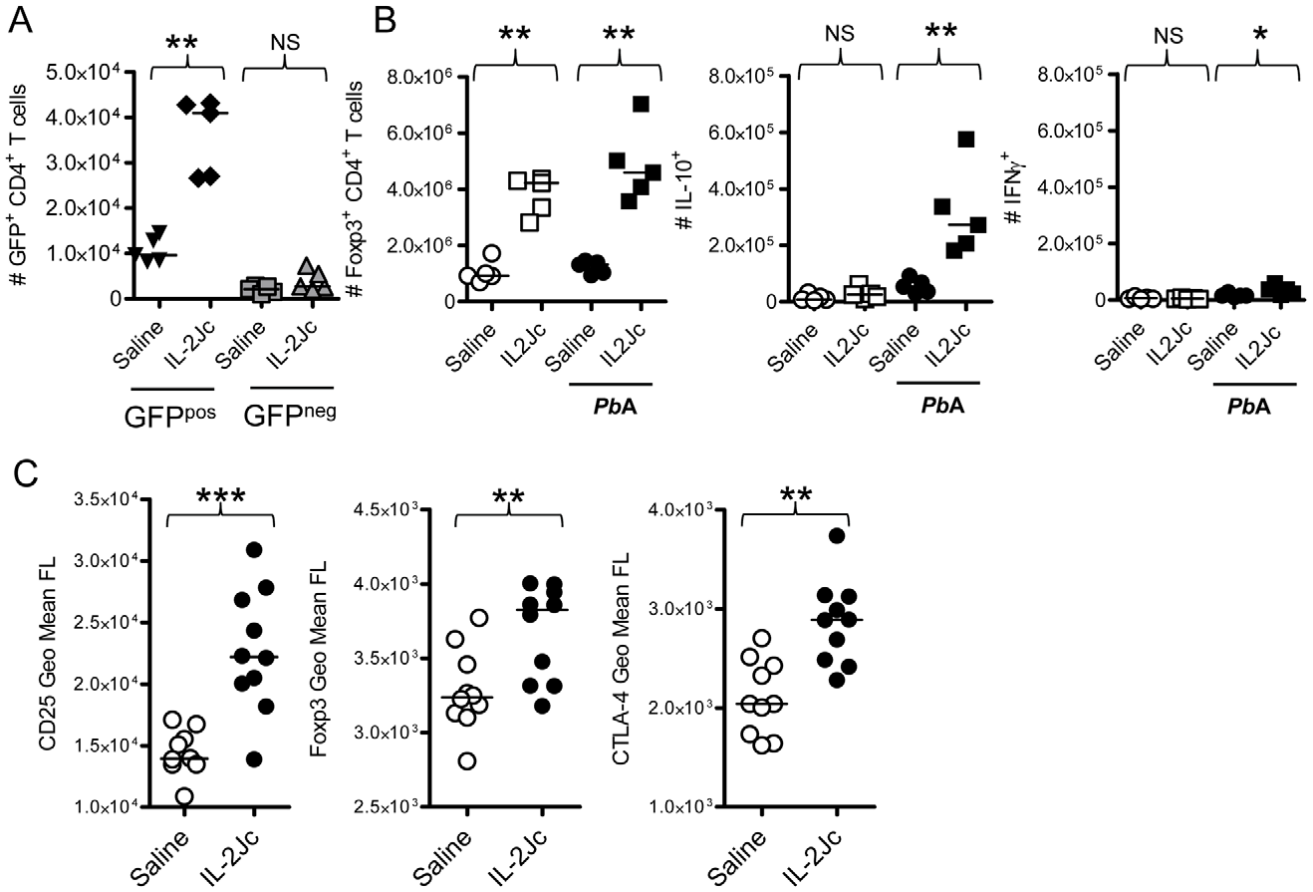
Analysis of Treg cells expanded by IL-2Jc during ECM. A) Viable Foxp3^+^ CD4^+^ Treg cells (GFP^pos^), and Foxp3^−^ CD4^+^ non-Treg cells (GFP^neg^) were cell sorted from the spleens of naïve *foxp3^gfp/gfp^* mice. 1×10^6^ GFP^pos^ cells or 5×10^6^ GFP^neg^ cells were adoptively transferred into mice, which were then infected with *Pb*A, and treated either with IL-2Jc or control saline. Four days later, numbers of splenic GFP^+^ CD4^+^ T cells were enumerated. B) Naïve C57BL/6 mice, and those infected with *Pb*A, were treated with IL-2Jc or control saline. Four days later, spleens were isolated, Foxp3^+^ CD4^+^ T cells were enumerated by flow cytometry, and IL-10 and IFNγ production by these cells was assessed directly ex vivo by intracellular cytokine staining. C) Four days after infection with *Pb*A, splenic CD4^+^ Foxp3^+^ T cells from IL-2Jc treated and control saline treated mice we assessed for expression of CD25, Foxp3 and CTLA-4 by flow cytometry. Mann-Whitney: ***p<0.001; **p<0.01; *p<0.05; NS-not statistically significant.

We next determined whether the increase in Treg cell numbers caused by IL-2Jc treatment during infection was the result of natural Treg cell expansion, or de novo conversion of naïve, Foxp3^−^ CD4^+^ T cells into Treg cells. Foxp3^+^ CD4^+^ natural Treg cells and Foxp3^−^ CD4^+^ T cells were sorted from the spleens of naïve *foxp3^gfp/gfp^* mice [41], and transferred into C57BL/6 mice. On the same day, these mice were infected, and treated either with IL-2Jc or saline. At the peak of Treg cell expansion (4 days later), splenic GFP^+^ Foxp3^+^ CD4^+^ Treg cells were enumerated. These cells were readily detected in mice that received GFP^+^ natural Treg cells, and indeed their numbers were boosted by IL-2Jc treatment (Figure 6A). However, in mice receiving GFP^−^ non-Treg CD4^+^ T cells, we observed no evidence of their conversion into Foxp3^+^ Treg cells either spontaneously during infection or after stimulation with IL-2Jc. These data indicate that IL-2Jc treatment during ECM triggers natural Treg cell expansion, but not conversion of Foxp3^−^ CD4^+^ T cells to a Foxp3^+^ phenotype.

We next examined whether natural Treg cell expansion caused by IL-2Jc was dependent upon infection. Naïve and infected groups of mice were treated with IL-2Jc or control saline. Four days later, the number of splenic Foxp3^+^ CD4^+^ T cells was determined (Figure 6B). Consistent with previous reports [23], [25], substantial Treg cell expansion was observed in both naïve and infected mice, demonstrating that this phenomenon is not dependent on infection. Importantly, however, when we assessed direct ex vivo production of cytokines by Treg cells (by intracellular cytokine staining with no in vitro stimulation) (Figure 6B), we noted that while expanded Treg cells in naïve mice made little IL-10, those in IL-2Jc treated, infected mice, made significantly higher amounts of this cytokine than those from control, infected mice. A small number of Treg cells from IL-2Jc-treated, infected mice, also appeared to make IFNγ, but this response was much lower than the IL-10 response (Figure 6B). These data indicate that IL-2Jc triggers the expansion of IL-10-producing Treg cells during *Pb*A infection. We further analysed the effects of IL-2Jc on Treg cells, and observed that their expression of CD25, Foxp3 and CTLA-4 was substantially elevated by IL-2Jc treatment compared to control saline treated, infected mice (Figure 6C). Taken together, these data demonstrate that IL-2Jc treatment triggers the expansion of natural CD4^+^ Treg cells, which then express higher levels of Foxp3, IL-10 and CTLA-4 in response to *Pb*A infection.

### IL-2Jc–mediated protection against ECM is dependent on Foxp3^+^ Treg cells, and CTLA-4, but not IL-10

**Figure 7:**
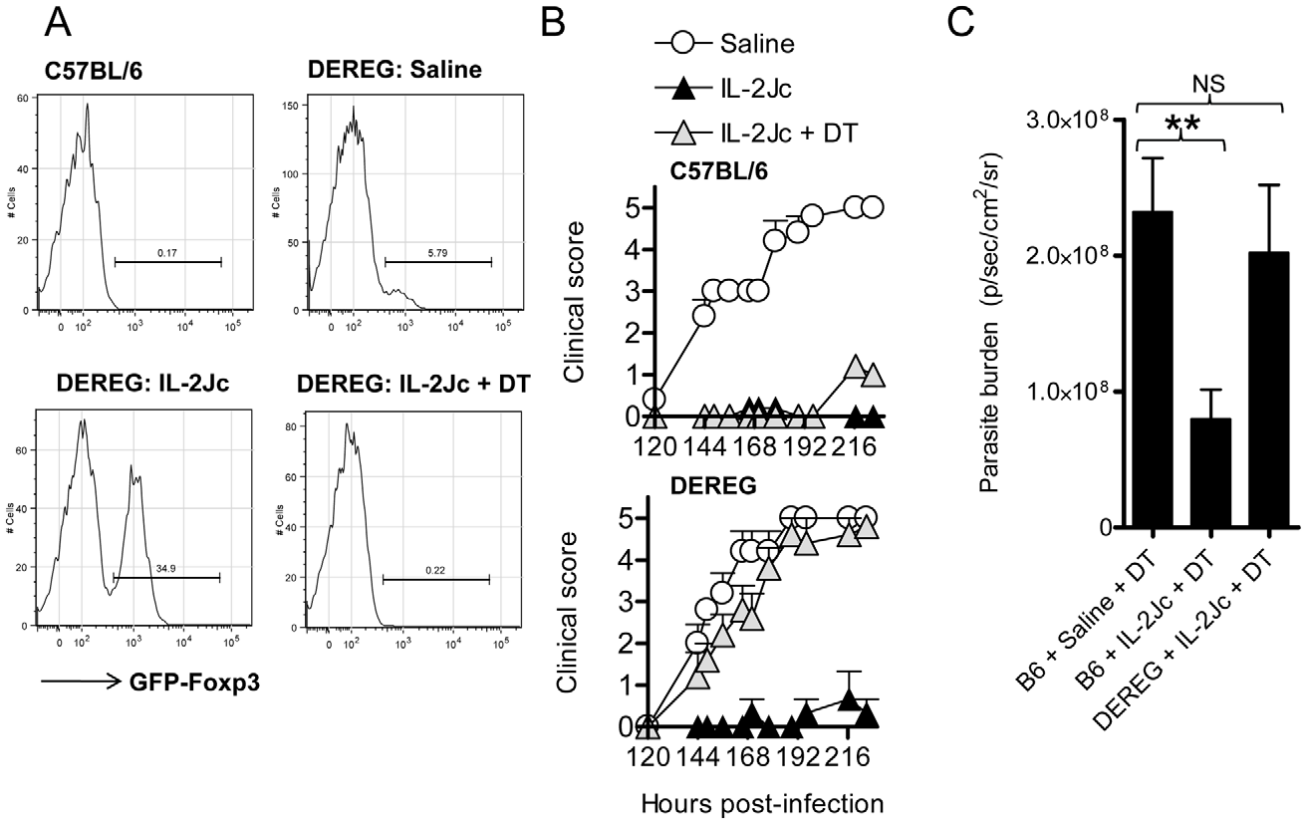
IL-2Jc-mediated protection against ECM is dependent on Foxp3^+^ cells. C57BL/6 and DEREG mice (n = 5–6) were *Pb*A-infected and treated with IL-2Jc or saline on the same day. IL-2Jc-treated C57BL/6 and DEREG mice were i.p. treated on days 3, 5, & 7 p.i. with 1µg of DT or saline. A) On day 4 p.i. blood CD4^+^ T cells were analysed by flow cytometry for the presence of GFP^+^ Foxp3^+^ Treg cells. B) Mice (C57BL/6: upper graph)(DEREG: lower graph) were monitored for clinical symptoms from day 5 p.i. (120 hours p.i.). These data are representative of 2 independent experiments. C) C57BL/6 mice and DEREG mice (n = 5–7) were IL-2Jc or control saline treated on the day of infection with *Pb*A. All mice were DT treated, and on day 6 p.i. whole body parasite burdens were assessed. Mann-Whitney: **p<0.01.

To determine if Treg cells were important for IL-2Jc mediated protection against ECM, we employed the DEREG mice [5]. C57BL/6 mice and DEREG mice were infected with *Pb*A, and treated with IL-2Jc or saline. DT or saline was administered to IL-2Jc treated DEREG and C57BL/6 mice from day 3 p.i., around the peak expansion of Treg cells. The following day (day 4 p.i.), while Treg cell expansion was evident in DEREG mice given IL-2Jc, depletion of Treg cells (>95% efficacy in this study) was confirmed in mice that had received DT (Figure 7A). C57BL/6 mice were completely protected from ECM when treated with IL-2Jc, either with or without DT treatment (Figure 7B), indicating no side-effects of DT treatment in C57BL/6 mice during *Pb*A-infection over this time-frame. DEREG mice were equally susceptible to ECM as C57BL/6 mice, and were protected by IL-2Jc treatment (Figure 7B). Crucially, IL-2Jc-mediated control of parasite burdens and protection from disease was completely abrogated when DEREG mice were treated with DT (Figure 7B & 7C). These data formally demonstrate that Foxp3^+^ cells are responsible for IL-2Jc-mediated protection against ECM.

**Figure 8:**
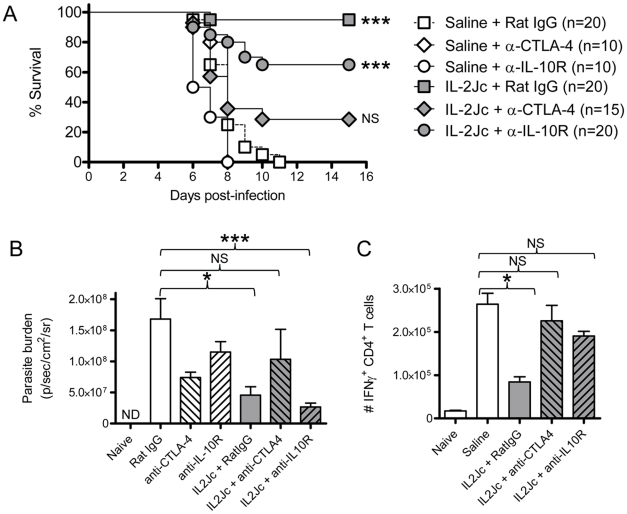
IL-2Jc-mediated protection against ECM is dependent on CTLA-4 more than IL-10. C57BL/6 mice (n = 10–20 per group) were *PbA*-infected, and treated with IL-2Jc or control saline. On days 3 and 5 p.i. mice were i.p. treated with 0.5mg of anti-CTLA-4 mAb, anti-IL-10R mAb, or control rat IgG antibodies. Mice were monitored for A) survival, B) whole body parasite burden on day 6 p.i., with some groups also being analysed for C) splenic IFNγ^+^ CD4^+^ T cell responses on day 4 p.i. Data in A) are pooled from 3 independent experiments, with statistical comparisons made relative to the “saline + Rat IgG” group. Log Rank tests performed in A), and Mann-Whitney tests in B) & C) ***p<0.001; *p<0.05; NS-not statistically significant.

Since IL-2Jc treatment increased IL-10 and CTLA-4 expression by Foxp3^+^ CD4^+^ T cells during infection (Figure 6B & 6C), we hypothesized that protection was dependent upon these two molecules. To test this, IL-2Jc-treated, *Pb*A-infected C57BL/6 mice received anti-CTLA-4 or anti-IL-10R blocking antibodies, or control IgG from day 3 p.i.. Anti-CTLA-4 significantly reduced IL-2Jc-mediated protection, with >60% of IL-2Jc-treated mice succumbing to infection with pathogen burdens similar to control infected mice (Figure 8A & 8B). Anti-IL-10R blockade, on the other hand, only partially reversed IL-2Jc-mediated protection, with >60% survival (Figure 8A), and interestingly, further reduced pathogen burdens in IL-2Jc treated mice (Figure 8B). Both antibody blockade treatments restored the splenic IFNγ CD4^+^ T cell response that had been impaired by IL-2Jc treatment (Figure 8C). Since we could detect only a modest role for IL-10 in IL-2Jc mediated protection of wild-type C57BL/6 mice, we further examined the effect of IL-2Jc treatment in IL-10^−/−^ mice, and found that these animals were significantly protected against ECM in a CTLA-4-dependent manner (Figure S4). These data demonstrate that IL-10 is not essential for IL-2Jc-expanded natural Treg cells to protect against ECM.

In addition, non-IL-2Jc treated mice were also treated with anti-CTLA-4 or anti-IL-10R blocking antibodies to study their effects alone on the course of ECM. Anti-CTLA-4 treatment did not alter disease outcome in saline treated mice (Figure 8A), despite partially reducing parasite burdens (Figure 8B), while anti-IL-10R treatment significantly accelerated ECM onset (Figure 8A) (MST 6.5 days vs. 8 days; p<0.01), with a partial reduction in parasite burden (Figure 8B). Of note, when anti-IL-10R mAb was administered from the start of infection, a more substantial reduction on parasite burden is observed [19].

Taken together, these data demonstrate that when CTLA-4 was blocked in IL-2Jc treated mice, pathogenic CD4^+^ T cell responses were restored, pathogen burdens were poorly controlled and protection from ECM was reversed. Thus IL-2Jc mediated protection against ECM is strongly dependent on Foxp3^+^ cells, and CTLA-4, but not IL-10.

## Discussion

T cell responses to infection are generally required for pathogen control, but can also contribute to disease. The roles of T cells in the pathogenesis of severe malaria syndromes, including CM, are unclear. Leukocytes have been observed in the brains of patients who have died from CM [42], [43], but their contribution to CM pathogenesis is not known. Studies in the ECM model show that T cells play a critical role in disease pathogenesis [16], [18], although the role of Treg cells in this model remains the subject of debate [20], [21], [22]. Data from this study and others [22] suggest that Treg cells do little to impact on ECM onset, and may in some cases exacerbate disease [20], [21]. Higher Treg cell frequencies have been associated with elevated blood parasitemia in human malaria patients [4], [44], [45], suggesting that Treg cells impair pathogen clearance during malaria. However, one report demonstrated that anti-CD25 treatment of ECM-resistant BALB/c mice increased the incidence of neurological symptoms during secondary *Pb*A challenge [46], suggesting Treg cells might be protective against ECM. Here, we report that Treg cells can protect against ECM following their expansion in vivo. Thus, while Treg cell responses during ECM are usually insufficient to control pathogenic T cells, and Treg cell ablation has no effect on pathogen burden or disease outcome, if present in large enough numbers, Treg cells can prevent disease. This is the first report to clearly show that CD4^+^ Foxp3^+^ Treg cells can play a direct protective role during experimental malaria infection. However, it is important to bear in mind that Treg-mediated protection was only achieved by treatment with IL-2Jc from the start of infection, and this therapeutic opportunity will not exist in human malaria patients. Thus, alternative approaches to rapidly expand Treg cell numbers in a clinical setting would have to be considered for therapeutic effect.

Previous data from our laboratory showed that anti-CD25 (PC61) monoclonal antibody treatment, partially depleted/blocked Treg cells (i.e.,, affecting only those cells expressing high levels of CD25), enhanced anti-parasitic CD4^+^ T cell responses, and reduced both parasite burden and ECM incidence [20]. These data were interpreted to mean that natural Treg cells normally impair pathogen clearance, and thus help to promote ECM. However, it is now clear from this work, and from another recent report, that total depletion of natural Treg cells does not protect against ECM [22]. The discrepancy between the outcome of partial and total Treg cell depletion in ECM are unresolved at present, but could be linked to the fact that anti-CD25 mAb-treated mice retain a population of CD25^lo^ Foxp3^+^ CD4^+^ T cells that can display plasticity in vivo [47], and might therefore contribute to protection from disease.

Pathogen control is clearly inefficient during ECM, with little evidence that T cells provide any protection against infection. To date, only NK cells have been reported to mediate some pathogen control during ECM [13]. However, NK cell depletion in IL-2Jc-treated mice did not prevent protection from ECM, indicating that these cells were not targets for IL-2Jc and did not contribute to enhanced parasite control. We recently showed that T cells promote the accumulation of pRBC in multiple tissue sites during *Pb*A infection, and that depletion of either CD4^+^ or CD8^+^ T cells to protect from ECM dramatically reduced parasite tissue sequestration [19]. Furthermore, lymphocyte-deficient B6.RAG1-deficient mice failed to develop ECM and had markedly reduced parasite burdens compared to control animals and B cell-deficient mice following *PbA* infection [19]. Therefore, in C57BL/6 mice, *PbA* tissue sequestration is promoted by host T cell responses, possibly by the conditioning host tissue endothelial cells via cytokines to allow binding of pRBC, as described by others [48].

**Figure 9:**
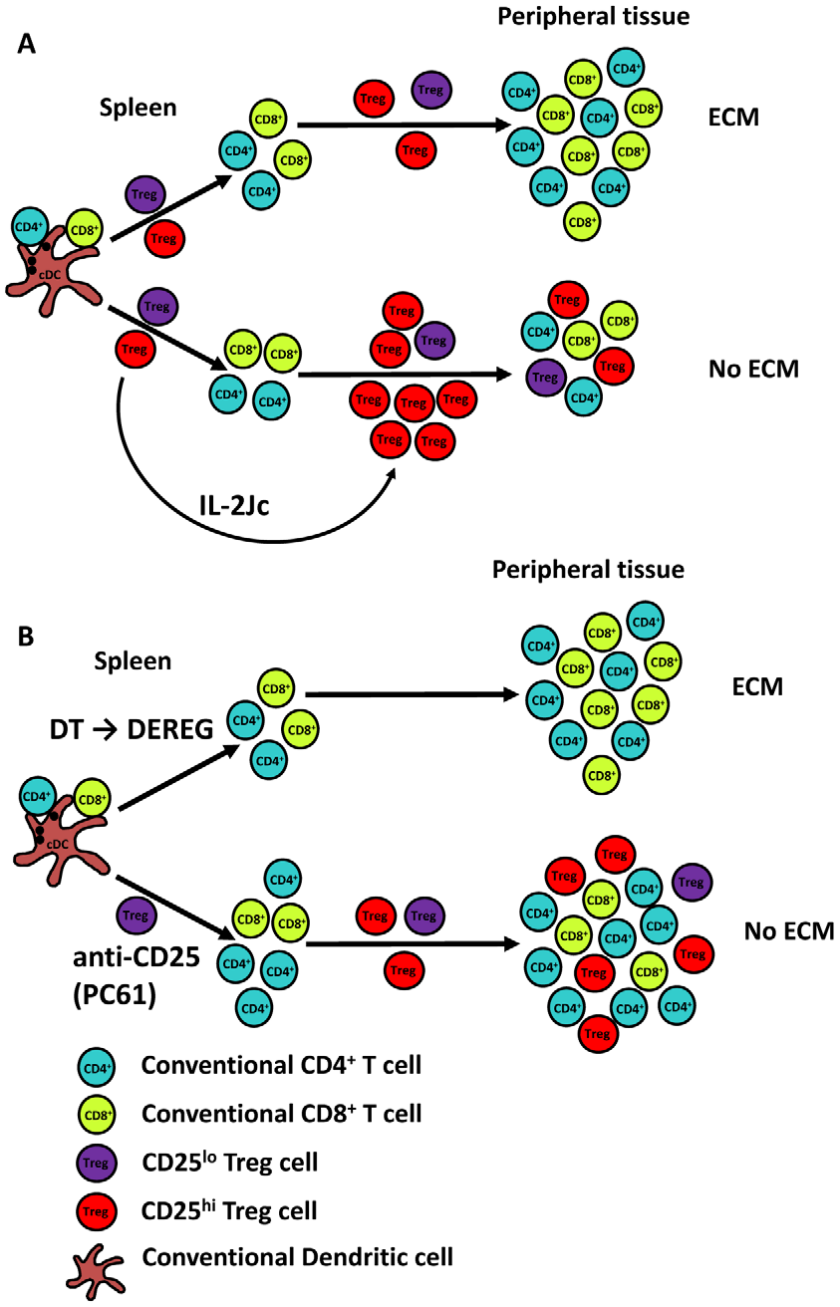
A proposed model for the roles of Treg cells in ECM. A). Following *PbA* infection of C57BL/6 mice, Treg cells suppress potent anti-parasitic T cells responses, but the *PbA*-specific T cell responses that do emerge promote parasite tissue sequestration, resulting in disease and death. However, if Treg cells can be expanded and activated sufficiently by early signalling via the high affinity IL-2 receptor, they can prevent pathogenic T cell responses mediating parasite tissue sequestration. B). The removal of CD25^hi^ Treg cells prior to *PbA* infection allows the generation of a potent anti-parasitic CD4^+^ T cell response, but also the re-emergence of CD25^hi^ Treg cells during the course of infection, resulting in an enhanced and qualitatively better anti-parasitic CD4^+^ T cells response that controls parasite growth and a mechanism to prevent parasite tissue sequestration. In contrast, if all Treg cells are depleted prior to infection, as is the case when DEREG mice are treated with DT, a balance between emerging anti-parasitic CD4^+^ T cell responses and expanding Treg cell responses is not achieved, and T cell-mediated immune pathology occurs. Conventional CD4^+^ (blue) and CD8^+^ (green) T cells, as well as CD25^hi^ (red) and CD25^lo^ or negative (purple) Treg cells are shown and disease outcome in different experimental conditions is shown.

Our earlier studies on anti-CD25 mAb treatment of *PbA*-infected mice indicated that early blockade/depletion of CD25^hi^ Treg cells allowed the generation of an enhanced anti-parasitic CD4^+^ T cell response that was accompanied by recovery and expansion of CD25^hi^ Treg cells during the course of infection [20]. Furthermore, our current data indicates that the most likely explanation for the protective effects of in vivo expanded Treg cells is the suppression of pathogenic T cell expansion that would otherwise promote parasite tissue sequestration. Thus, we propose a model whereby naturally occurring CD25^hi^ Treg cells suppress the development of potent anti-parasitic immunity early during *PbA* infection and their depletion/blockade results in enhanced anti-parasitic CD4^+^ T cells responses, reducing both parasite burdens and the risk of severe pathology. However, there also appears to be an important role for the remaining CD25^lo^ Foxp3^+^ CD4^+^ T cells in achieving a balance between emerging anti-parasitic immunity and immune-pathology in anti-CD25mAb-treated mice, as indicated by the failure of DT-mediated Treg cell depletion in DEREG mice to protect against ECM. A role for IL-10-producing inducible regulatory T cells [40] and/or IL-10/IFNγ-producing Th1 cells [4] identified in *P. yoelii*-infected mice and malaria patients, respectively, may also contribute to this latter process. Our data reported in this study shows that if Treg cells can be expanded sufficiently via IL-2Jc during *PbA* infection, they can suppress normally pathogenic T cell responses, and prevent parasite tissue sequestration and ECM. Hence, Treg cells could have two potentially important roles during *PbA* infection (Figure 9). First, they could suppress the early generation of anti-parasitic CD4^+^ T cells responses that are detrimental to the host, and second, they can modulate pathogenic T cell responses to reduce parasite tissue sequestration later during infection. This latter effect may allow better clearance of parasites in the spleen, thus lowering parasite burden. Again, we do not rule out the possibility that inducible regulatory T cells may also play a role in protecting against disease later in infection, and even restricting parasite tissue sequestration.

Treg cell-mediated protection against ECM was exquisitely sensitive to the timing of IL-2Jc treatment. Delaying treatment by 48 hours caused the selective expansion of conventional CD4^+^ T cells rather than Treg cells, presumably due to infection-induced expression of high affinity IL-2 receptor by the former cell population. However, this expansion of conventional CD4^+^ T cells was unable to control parasite growth and failed to protect from ECM. Treg cell depletion from day 3 p.i., completely abrogated IL-2Jc-mediated protection, showing that IL-2Jc-expanded Treg cells were responsible for protection from ECM. Clearly, there is a fine balance between the activation and expansion of anti-parasitic T cell responses and the emergence of disease-protective Treg cells that determines the outcome of *PbA* infection. Whether such an intimate relationship between these two different types of T cells exists during human malaria remains to be determined. Nevertheless, our data suggests a more complex temporal and spatial relationship between emerging anti-parasitic T cell responses required for control of parasite growth and the functions of Treg cells that may protect against disease, than has previously been recognised.

Treg cells function via multiple mechanisms, including CTLA-4 [11], IL-10, TGFβ and IL-2-deprivation [10]. We found that Treg cell-mediated protection against ECM required CTLA-4, but was only modestly affected by IL-10 blockade. Previous reports have demonstrated that a murine AIDS infection induces IL-10 expressing Treg cells, and that enhanced IL-10 levels protect against ECM [49], [50]. Our data is consistent with a moderate therapeutic role for IL-10 in ECM, but demonstrates that CTLA-4 is a more potent regulator of pathogenic T cell responses. We attempted to determine the antigen specificity of the IL-10 response made by Tregs cells in IL-2Jc treated mice, by sorting these cells, stimulating with APC and parasite antigen, and looking for IL-10 production at both the protein and mRNA level. However, these experiments are notoriously difficult to perform [51], [52], [53], and we were unable to assess the antigen specificity of IL-10 producing Tregs. There may also be different roles for the major regulatory molecules produced by Treg cells in our study (CTLA-4) and inducible regulatory T cells identified by others (IL-10; [4], [40]), by acting on different cellular/tissue targets during malaria. However, there is no direct evidence for this as yet.

In conclusion, we and others have shown that Treg cells numbers expand during malaria infection, but are unable to protect against T cell mediated immune pathology [20], [21], [40]. Here we show for the first time that Treg cells can protect against T cell-mediated immune pathology in malaria if their numbers are sufficiently expanded at the appropriate time during the immune response. Thus, while increased Treg cell frequencies may contribute to increased parasitemia in malaria patients [4], [44], [45], a further possibility is that these cells expand in an attempt to protect against disease caused by parasite sequestration.

## Materials and Methods

### Mice

Female C57BL/6 mice and congenic CD45.1^+^ C57BL/6 mice aged 6–8 weeks were purchased from the Australian Resource Centre (Canning Vale, Perth, Western Australia) and maintained under conventional conditions. DEREG mice [5], OTI [54], C57BL/6 *il10* ^−/−^, and C57BL/6 *Jalpha18^−/−^* mice were bred and maintained in house. *foxp3^gfp/gfp^* mice [41] were backcrossed ten times onto the C57BL/6 background, bred and maintained in house.

### Ethics statement

All animal procedures were approved and monitored by the Queensland Institute of Medical Research Animal Ethics Committee. This work was conducted under QIMR animal ethics approval number A02-633M, in accordance with the “Australian code of practice for the care and use of animals for scientific purposes” (Australian National Health & Medical Research Council).

### Parasites and infections

*P. berghei* ANKA (*Pb*A) strains were used in all experiments after one in vivo passage in mice. A transgenic *Pb*A (231c1l) clonal line expressing luciferase and green fluorescent protein under the control of the EF1-α promoter (*PbA*-luc) was used for all experiments unless stated otherwise [20]. Transgenic *Pb*A strains expressing model T cell epitopes, and control strains, *Pb*TG and *PbG*, were obtained from Prof. William R. Heath, University of Melbourne, Australia, and were maintained and used as previously reported [35]. All mice were infected with 10^5^ pRBCs intravenously (i.v.) via the lateral tail vein. Blood parasitemia was monitored by examination of Diff-Quick (Lab Aids, Narrabeen, NSW, Australia) stained thin blood smears obtained from tail bleeds.

### Disease assessment

Mice were monitored twice daily after day 5 p.i., and clinical ECM evaluated. Clinical ECM scores were defined by the presentation of the following signs: ruffled fur, hunching, wobbly gait, limb paralysis, convulsions, and coma. Each sign was given a score of 1. Animals with severe ECM (accumulative scores = 4) were sacrificed by CO_2_ asphyxiation according to ethics guidelines, and the following timepoint given a score of 5 to denote death.

### Antibodies and other reagents

Allophycocyanin (APC) or Pacific Blue (PB)-conjugated anti-TCRβ chain, phycoerythrin(PE)-Cy5- or PE-conjugated anti-CD4, PE-Cy5-conjugated anti-CD8, PE or fluorescein isothiocyanate-conjugated anti-CD45.1, APC or PE-conjugated anti-IFNγ, and PE-conjugated anti-IL-10 were purchased from Biolegend (San Diego,CA) or BD Biosciences (Franklin Lakes, NJ). Alexa-647-labelled anti-mouse Foxp3 mAb was purchased from eBioscience (San Diego, CA). PE-conjugated anti-human Granzyme B (GzmB), with mouse cross reactivity, was purchased from Invitrogen (Mount Waverley, Vic., Australia). Anti-CTLA-4 (UC10-4F10-11) and control IgG was purchased from BioXCell, (West Lebanon, NH, USA). Anti-IL-10R (1B1.3a), anti-CD4 (YTS191), anti-IL-2 (S4B6 and JES6-1A12), anti-NK1.1 (PK136), and isotype control mAb (MAC49; ratIgG1) were purified from culture supernatants by protein G column purification (Amersham, Uppsala, Sweden) followed by endotoxin removal (Mustang Membranes; PallLife Sciences, East Hills, NY). Purified control rat IgG were also used in some experiments and purchased from Sigma-Aldrich (Castle Hill, NSW, Australia). Diphtheria toxin (DT) was purchased from Sigma-Aldrich, diluted in saline, and 1µg doses injected via the intraperitoneal route.

### Preparation and in vivo administration of IL-2/anti–IL-2 complexes

1.5µg of recombinant murine IL-2 (eBioscience, San Diego, CA) was incubated with 50µg of either S4B6 or JES6-1A12 (prepared as detailed above) in saline, for 30 minutes at 37°C prior to intraperitoneal administration to each mouse in a volume of 200µl.

### Preparation of tissue mononuclear cells

Blood mononuclear cells were analysed in heparinised blood after 2 rounds of red cell lysis using hypotonic red cell lysis buffer according to the manufacturer's instructions (Sigma-Aldrich). Spleen cells were isolated by passing tissue through a 100-µm sieve in RPMI-1640 tissue culture medium supplemented with 2% (v/v) fetal calf serum (Wash Buffer). Red blood cells were lysed as above (Sigma-Aldrich) and washed once more with Wash Buffer. Brain mononuclear cells were isolated by digesting tissue in collagenase type 4 (1 mg/ml; Worthington Biochemical Corp., Lakewood, NJ) and deoxyribonuclease I (0.5 mg/ml; Worthington Biochemical) at room temperature for 40 minutes, before passing through a 100-µm sieve and washing twice with Wash Buffer. The cell pellet was resuspended in 33% (v/v) Percoll in PBS and centrifuged at 693×*g* for 12 minutes at room temperature. Supernatant containing debris was removed, and the leukocyte pellet was washed once in Wash Buffer, red blood cells lysed as described above, and washed and resuspended in RPMI-1640 medium supplemented with 5% (v/v) fetal calf serum.

### Flow cytometric analysis

For the staining of cell surface antigens, cells were incubated with fluorochrome-conjugated mAbs on ice for 20 minutes. Intracellular staining for Foxp3 was performed on fixed/permeabilized cells using Alexa647-labeled anti-mouse Foxp3 kit (eBioscience), according to the manufacturer's instructions. Intracellular cytokine staining for IFNγ, CTLA-4 and GzmB was performed using a BD Fixation/Permeabilisation kit (BD Biosciences) according to manufacturer's instructions. Data were acquired on a FACSCanto II flow cytometer (BD Biosciences) and analysed using FlowJo software (Treestar, Ashland, OR, USA). Cell populations in the blood, spleen and brain were defined as follows: CD4^+^ T cells (CD4^+^TCRβ^+^), CD8^+^T cells (CD8α^+^TCRβ^+^), NK cells (NK1.1^+^TCRβ^−^), CD4^+^ Treg cells (CD4^+^Foxp3^+^TCRβ^+^). Cytokines in tissue culture supernatants and serum samples were quantified using the cytometric bead array flexsets (BD Biosciences) on a FACSarray equipped with BD Flexset analysis software (BD Biosciences).

### Ex vivo antigen restimulation of CD4+ T cells

Splenic CD4^+^ T cells (5×10^4^ cells/well), either bulk populations purified to >85% purity by magnetic bead positive selection techniques (Miltenyi Biotec; North Ryde, NSW, Australia), or cell sorted to isolate Treg cells from *foxp3^gfp/gfp^* mice to a purity >99%, were stimulated with 2.5×10^5^ PbA-parasitized RBC (pRBC) or naive RBC (nRBC), and 1×10^6^ irradiated naive C57BL/6 spleen cells at 37°C in 5% (v/v) CO_2_. Cell culture supernatant was collected after 24h or 72 h and cytokines were measured as above (BD Biosciences). After 72 h of culture, cells were pulsed with 1 µCi [^3^H]thymidine for 18 h, before measuring thymidine incorporation using a Betaplate Reader (Wallac).

### In vivo bioluminescence imaging

Luciferase-expressing *Pb*A pRBCs were visualized by imaging whole bodies or dissected organs with an I-CCD photon-counting video camera and in vivo imaging system (IVIS 100; Xenogen, Alameda, CA). Mice were anesthetized with isofluorane and injected intraperitoneally with 0.1 ml of 5 mg/ml D-luciferin firefly potassium salt (Xenogen). 5 minutes afterwards, images were captured on the IVIS 100 according to the manufacturer's instructions. Parasites were visualized in the brain after removal from mice that had been perfused with 15ml of saline via the heart. Bioluminescence generated by luciferase transgenic *Pb*A in mice or brain tissue was measured according to the manufacturer's instructions. The unit of measurement was photons/second/cm^2^/steer radiant (p/sec/cm^2^/sr).

### Statistical analysis

Differences in survival of treatment groups were analysed using the Kaplan-Meier log-rank test. All other analyses of differences in parasitemia, cytokine levels, cell numbers, bioluminescence etc. were performed using the Mann-Whitney nonparametric test. For all statistical tests, p<0.05 was considered significant. In all figures, *, **, *** denote p values of p<0.05, p<0.01 & p<0.001 respectively.

## Supporting Information

Figure S1 **IL-2Jc-mediated protection against ECM is independent of NK cells and invariant NKT cells.** A) C57BL/6 mice (n = 5) were infected and treated with IL-2Jc, IL-2Sc or control saline for 24 hours. Splenic NK cells (NK1.1^+^ TCR^−^) and NKT cells (NK1.1^+^ TCR^+^) were assessed directly ex vivo for expression of CD69, GzmB and IFNγ by flow cytometry. Mann-Whitney tests performed relative to saline-treated, infected mice: **p<0.01; NS = not statistically significant. B & C) C57BL/6 wild-type mice (n = 5), treated with anti-NK1.1 or control IgG (0.5mg i.p on days −2, +1 and +4 p.i.), and *Jα18^−/−^* mice (n = 5) were infected and treated with IL-2Jc or control saline. B) The percentage of CD4^+^ T cells in the blood expressing Foxp3 was determined on Day 4 p.i. C) Parasite burdens were determined on Day 6 p.i. Mann-Whitney tests performed relative to each saline-treated control group: **p<0.01 *p<0.05.
(0.09 MB TIF)

Figure S2 **IL-2Jc blocks serum IFNγ production during ECM.** Serum from naïve C57BL/6 mice (n = 5), and *PbA*-infected and variously treated mice was assessed for IFNγ on day 4 p.i.; Mann-Whitney: **p<0.01. This data is representative of 3 independent experiments.
(0.14 MB TIF)

Figure S3 **Ex vivo CD4^+^ T cell antigen-specific recall responses are reduced in IL-2Jc treated mice**: On day 4 p.i., CD4^+^ splenocytes were isolated from individual naïve, *PbA*-infected & saline treated, and *PbA*-infected & IL-2Jc or IL-2Sc treated C57BL/6 mice (n = 5). A) Cells from individual mice were stimulated with parasitized RBC (pRBC), or non-parasitized RBC (nRBC) and the ratio of antigen-specific proliferation in response to pRBC relative to nRBC was determined per mouse. B) Supernatants from stimulated CD4^+^ spleen cells were assessed for IFNγ levels. Mann-Whitney: ** p<0.01; *** p<0.001.
(0.48 MB TIF)

Figure S4 **IL-10 is not essential for IL-2Jc mediated protection against ECM.** IL-10^−/−^ C57BL/6 mice (n = 10–20) were infected with *Pb*A, and immediately treated with IL-2Jc or control saline. On days 3 & 5 p.i., IL-2Jc treated IL-10^−/−^ mice were injected i.p. with 0.5mg of anti-CTLA-4 or control IgG. Mice were monitored for survival. Statistical analyses indicate comparison with saline treated mice: Log Rank: ** p<0.01; *** p<0.001. Data is pooled from 2 independent experiments.
(0.10 MB TIF)
