# Supplementary figures and images for: CD4+ Natural Regulatory T Cells Prevent Experimental Cerebral Malaria via CTLA-4 When Expanded In Vivo
Source: PLoS Pathog. 2010 Dec 9;6(12):e1001221. doi: 10.1371/journal.ppat.1001221 (PMC3000360; doi:10.1371/journal.ppat.1001221)

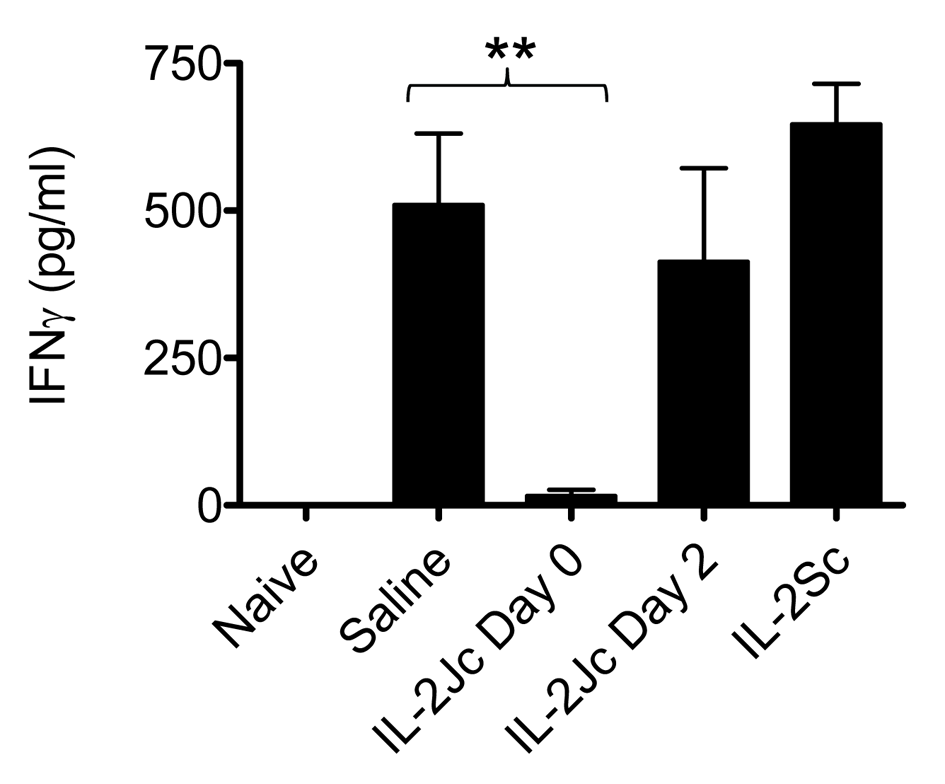

Supplement: Figure S1 — IL-2Jc-mediated protection against ECM is independent of NK cells and invariant NKT cells. A) C57BL/6 mice (n = 5) were infected and treated with IL-2Jc, IL-2Sc or control saline for 24 hours. Splenic NK cells (NK1.1+ TCR−) and NKT cells (NK1.1+ TCR+) were assessed directly ex vivo for expression of CD69, GzmB and IFNγ by flow cytometry. Mann-Whitney tests performed relative to saline-treated, infected mice: **p<0.01; NS = not statistically significant. B & C) C57BL/6 wild-type mice (n = 5), treated with anti-NK1.1 or control IgG (0.5mg i.p on days −2, +1 and +4 p.i.), and Jα18−/− mice (n = 5) were infected and treated with IL-2Jc or control saline. B) The percentage of CD4+ T cells in the blood expressing Foxp3 was determined on Day 4 p.i. C) Parasite burdens were determined on Day 6 p.i. Mann-Whitney tests performed relative to each saline-treated control group: **p<0.01 *p<0.05. (0.09 MB TIF) [file ppat.1001221.s001.tif]

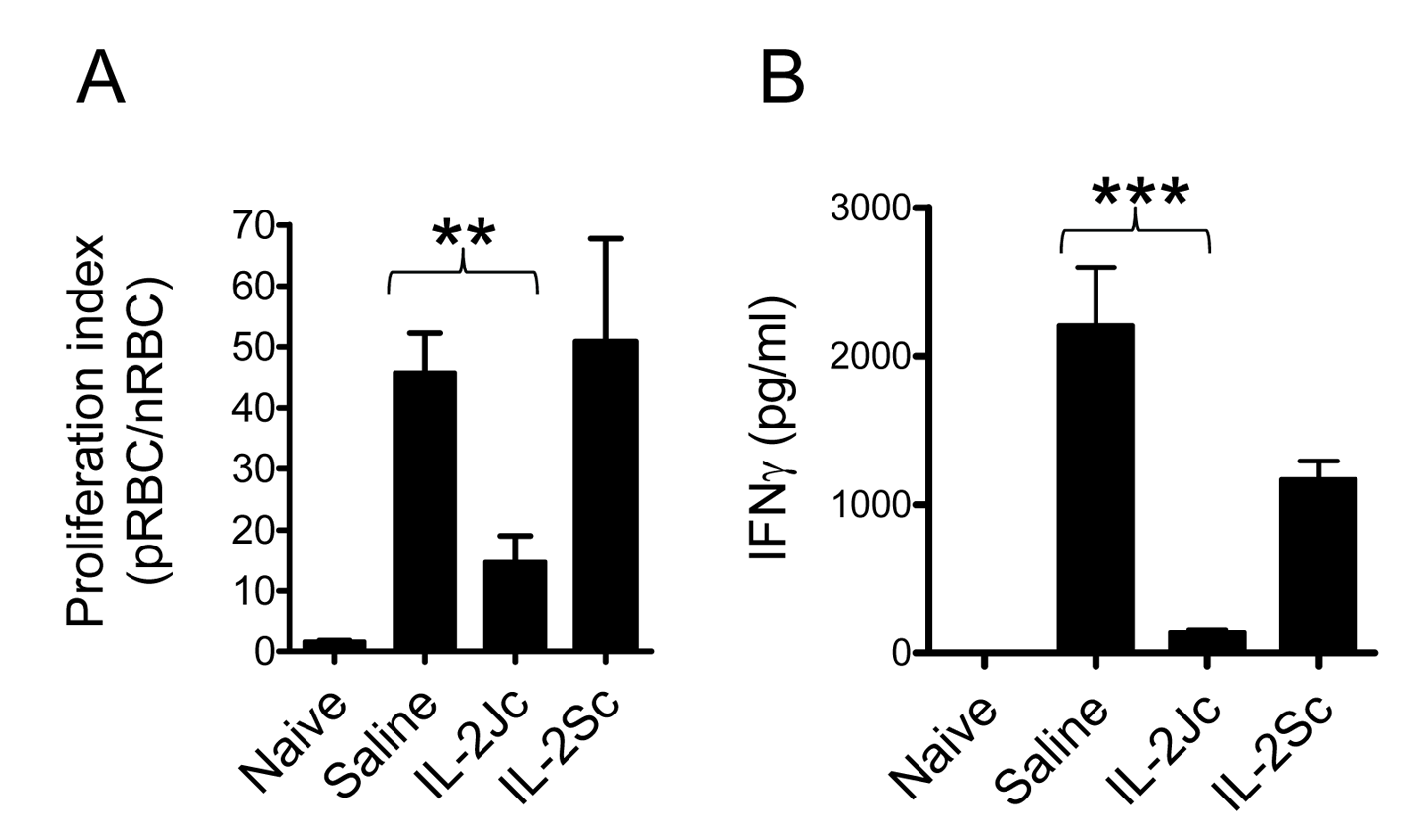

Supplement: Figure S2 — IL-2Jc blocks serum IFNγ production during ECM. Serum from naïve C57BL/6 mice (n = 5), and PbA-infected and variously treated mice was assessed for IFNγ on day 4 p.i.; Mann-Whitney: **p<0.01. This data is representative of 3 independent experiments. (0.14 MB TIF) [file ppat.1001221.s002.tif]

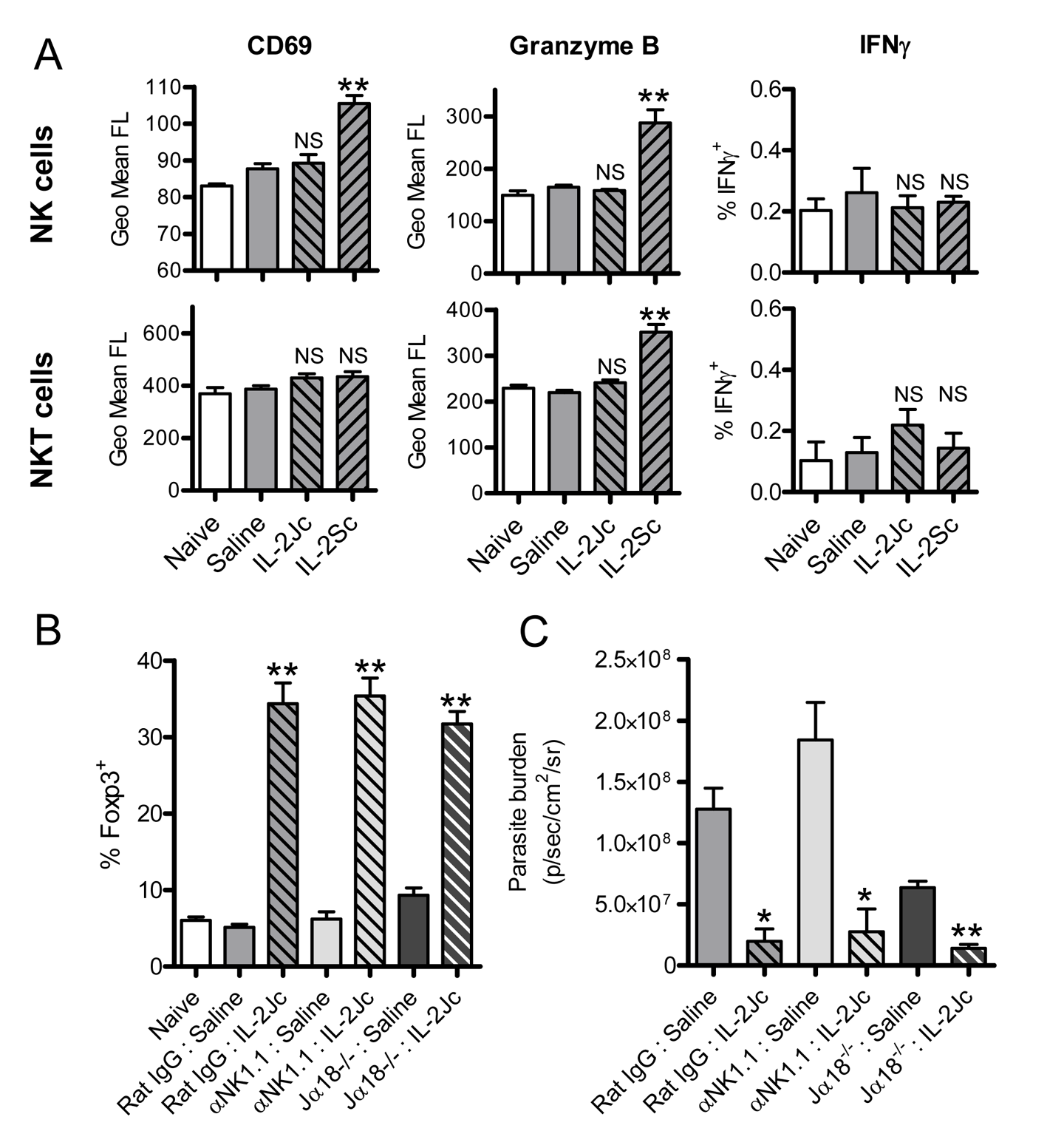

Supplement: Figure S3 — Ex vivo CD4+ T cell antigen-specific recall responses are reduced in IL-2Jc treated mice: On day 4 p.i., CD4+ splenocytes were isolated from individual naïve, PbA-infected & saline treated, and PbA-infected & IL-2Jc or IL-2Sc treated C57BL/6 mice (n = 5). A) Cells from individual mice were stimulated with parasitized RBC (pRBC), or non-parasitized RBC (nRBC) and the ratio of antigen-specific proliferation in response to pRBC relative to nRBC was determined per mouse. B) Supernatants from stimulated CD4+ spleen cells were assessed for IFNγ levels. Mann-Whitney: ** p<0.01; *** p<0.001. (0.48 MB TIF) [file ppat.1001221.s003.tif]

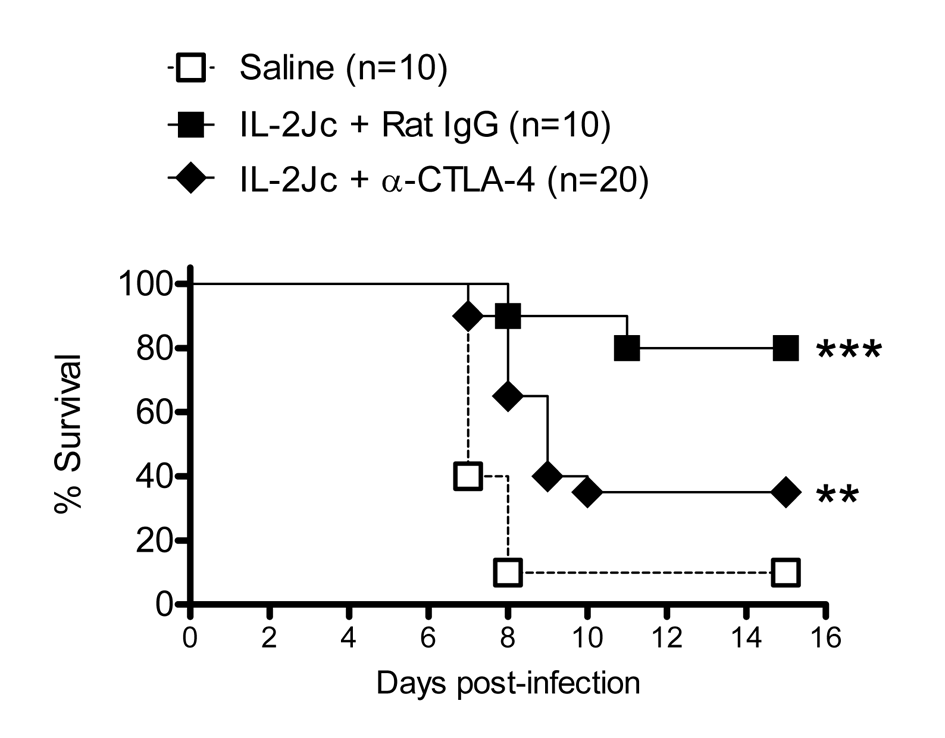

Supplement: Figure S4 — IL-10 is not essential for IL-2Jc mediated protection against ECM. IL-10−/− C57BL/6 mice (n = 10–20) were infected with PbA, and immediately treated with IL-2Jc or control saline. On days 3 & 5 p.i., IL-2Jc treated IL-10−/− mice were injected i.p. with 0.5mg of anti-CTLA-4 or control IgG. Mice were monitored for survival. Statistical analyses indicate comparison with saline treated mice: Log Rank: ** p<0.01; *** p<0.001. Data is pooled from 2 independent experiments. (0.10 MB TIF) [file ppat.1001221.s004.tif]
